# Improving Self-Management Skills Among People With Spinal Cord Injury: Protocol for a Mixed-Methods Study

**DOI:** 10.2196/11069

**Published:** 2018-11-14

**Authors:** W Ben Mortenson, Patricia Branco Mills, Jared Adams, Gurkaran Singh, Megan MacGillivray, Bonita Sawatzky

**Affiliations:** 1 Department of Occupational Sciences and Occupational Therapy Faculty of Medicine University of British Columbia Vancouver, BC Canada; 2 GF Strong Rehabilitation Centre Vancouver, BC Canada; 3 International Collaboration on Repair Discoveries Vancouver, BC Canada; 4 Physical and Rehabilitation Medicine Faculty of Medicine University of British Columbia Vancouver, BC Canada; 5 Self Care Catalysts Inc Toronto, ON Canada; 6 Rehabilitation Sciences Faculty of Medicine University of British Columbia Vancouver, BC Canada; 7 Department of Orthopaedics Faculty of Medicine University of British Columbia Vancouver, BC Canada

**Keywords:** self-management, spinal cord injury, eHealth, mHealth

## Abstract

**Background:**

Most people with spinal cord injury will develop secondary complications with potentially devastating consequences. Self-management is a key prevention strategy for averting the development of secondary complications and their recurrence. Several studies have shown that self-management programs improve self-management behaviors and health outcomes in individuals living with chronic conditions such as asthma, diabetes, hypertension, and arthritis. Given the burgeoning health care costs related to secondary complications, we developed an alternative electronic health–based implementation to facilitate the development of self-management skills among people with spinal cord injury.

**Objective:**

This study aims to evaluate the efficacy of a self-management app in spinal cord injury populations. The primary outcome is attainment of self-selected, self-management goals. Secondary outcomes include increases in general and self-management self-efficacy and reductions in self-reported health events, health care utilization, and secondary complications related to spinal cord injury. This study also aims to explore how the intervention was implemented and how the app was experienced by end users.

**Methods:**

This study will employ a mix of qualitative and quantitative methods. The quantitative portion of our study will involve a rater-blinded, randomized controlled trial with a stepped wedge design (ie, delayed intervention control group). The primary outcome is successful goal attainment, and secondary outcomes include increases in self-efficacy and reductions in self-reported health events, health care utilization, and secondary conditions related to spinal cord injury. The qualitative portion will consist of semistructured interviews with a subsample of the participants.

**Results:**

We expect that the mobile self-management app will help people with spinal cord injury to attain their self-management goals, improve their self-efficacy, reduce secondary complications, and decrease health care utilization.

**Conclusions:**

If the results are positive, this study will produce credible new knowledge describing multiple outcomes that people with spinal cord injury realize from an app-based self-management intervention and support its implementation in clinical practice.

**Trial Registration:**

ClinicalTrials.gov NCT03140501; http://clinicaltrials.gov/ct2/show/NCT03140501 (Archived by WebCite at http://www.webcitation.org/73Gw0ZlWZ)

**International Registered Report Identifier (IRRID):**

PRR1-10.2196/11069

## Introduction

### Background

Damage to the spinal cord following injury can cause sensory, motor, and autonomic impairments that lead to serious and sometimes fatal secondary complications. The prevalence of spinal cord injury in the United States is estimated at 906 per million [1]. Most spinal cord injuries in young adults are attributable to traffic accidents and sports injuries [2], whereas the most common cause of spinal cord injury in older adults is falls [3].

Most people with spinal cord injury will develop secondary complications [4]. For example, during a yearly medical check-up, more than 95% of people with spinal cord injury reported experiencing at least 1 secondary complication associated with their spinal cord injury and 58% reported experiencing 3 or more complications [5]. Some of the most common secondary complications in community-dwelling individuals with spinal cord injury include autonomic dysreflexia, depression, renal problems, and pressure ulcers [6,7].

Secondary complications associated with spinal cord injury can have devastating and costly consequences. For example, individuals experiencing pressure ulcers are often prescribed prolonged bed rest, which can prevent them from participating in community activities [8]. This may result in a negative feedback loop as social isolation can lead to depression, and physical inactivity may lead to weight gain and deconditioning. In extreme cases, pressure ulcers may be fatal if the wound becomes infected with an antibacterial-resistant microorganism [9]. It generally takes about 23 days to heal a pressure ulcer with a direct cost of US $1971 for a stage I (shallow) ulcer and US $19,554 for a stage IV (deep) ulcer [10,11]. It has been estimated that, on average, pressure ulcer complications can add US $43,180 to a hospital stay in the United States [12].

Self-management is a key prevention strategy for averting the development and recurrence of secondary complications [13]. Self-management has been defined as “the individual’s ability to manage the symptoms, treatment, physical and psychological consequences and lifestyle changes inherent in living with a chronic condition” [14]. Self-management programs are aimed at increasing one’s problem-solving and decision-making skills [15]. Interventions to improve self-management are typically informed by social cognitive theory, which has 4 main tenets [16]: self-observation, self-evaluation, self-reaction, and self-efficacy (ie, belief in one’s ability to perform tasks and obtain goals) [16]. According to this theory, self-efficacy can be fostered via experiences of mastery, social modeling, social persuasion, and positive interpretation of physiological reactions [16]. This suggests that learning will be most effective when individuals are given tasks that are appropriate to their experience level, when they are relaxed and confident, and when they are encouraged to achieve the desired outcome [16,17].

Self-management may be an important strategy to improve health outcomes for individuals living with chronic conditions such as spinal cord injury. Several studies have shown that self-management programs improve self-management behaviors and health outcomes in individuals living with chronic conditions such as asthma, diabetes, hypertension, and arthritis [14,18,19]. For example, Barlow et al found that self-management interventions tailored for specific chronic conditions increased self-management behaviors such as monitoring blood glucose in diabetes, managing medication and symptoms in asthma, and managing psychosocial consequences and lifestyle changes in arthritis [11].

With rising health care costs and a shortage of qualified personnel for in-person interventions, more economical ways to teach self-management interventions are being explored, such as the use of eHealth interventions [20]. eHealth interventions may be advantageous over traditional approaches because they (1) are accessible to more people including those who live outside of large urban centers, (2) can be delivered simultaneously to a larger number of individuals, and (3) may be more cost-effective than the traditional face-to-face self-management interventions [21,22]. Evidence from studies involving individuals with diabetes and cigarette smokers show that the most effective technology-based self-management interventions are adaptable and specifically tailored to the end users [23,24]. Azar et al demonstrated that periodic and sustained engagement of the individual is crucial to support lasting behavioral change when using a mobile phone app for weight management [25]. A meta-analysis of the effectiveness of Web-based interventions over non-Web-based interventions for chronic disease management reported increases in knowledge of the condition, participation in health care, maintenance of behavioral changes, and slower health decline of older participants [22]. More recent studies on technology-based interventions corroborate these early findings. For example, eHealth self-management interventions involving Web-based self-monitoring of personal health have been found to improve diabetes management [25-28].

Despite the potential benefits of self-management strategies among people with spinal cord injury, we were able to identify little experimental research that has been conducted in this area. A study on a self-management program for people with indwelling urinary catheters found that participants who used these strategies decreased the frequency of complications associated with catheters [29]. A scoping review of technological interventions that support self-management of pressure ulcer (eg, computer-based educational technologies and telemedicine programs) found that these technologies demonstrated low-to-moderate effectiveness in reducing risk factors associated with pressure ulcer [30].

We have developed a mobile app designed to facilitate self-management behavior and skill development following spinal cord injury in the inpatient rehabilitation and early community reintegration. This broad-based, mobile self-management app was developed with the input of key stakeholders including people with spinal cord injury and their formal and informal caregivers [31].

### Study Objectives

The overarching objective of the study is to evaluate the efficacy of a self-management intervention that features the use of a self-management app among community-dwelling individuals with spinal cord injury who were discharged at least 12 months after inpatient rehabilitation. The primary outcome is attainment of self-selected, self-management goals. Secondary outcomes include increases in general and self-management self-efficacy and reductions in self-reported health outcomes, health care utilization, and secondary complications related to spinal cord injury. We will also examine how the intervention was implemented and how the app was experienced by end users.

We hypothesize that community-dwelling individuals with spinal cord injury who receive our self-management intervention, which features a self-management app, will have significantly better attainment of self-selected, self-management goals than those in the delayed control group.

### Ethics Approval

Ethical approval was obtained from the University of British Columbia’s Behavioral Research Ethics Board and the Vancouver Coastal Health Research Institute. Furthermore, the study has been prospectively registered before the first patient was enrolled in the study.

## Methods

### Study Design

**Figure 1:**
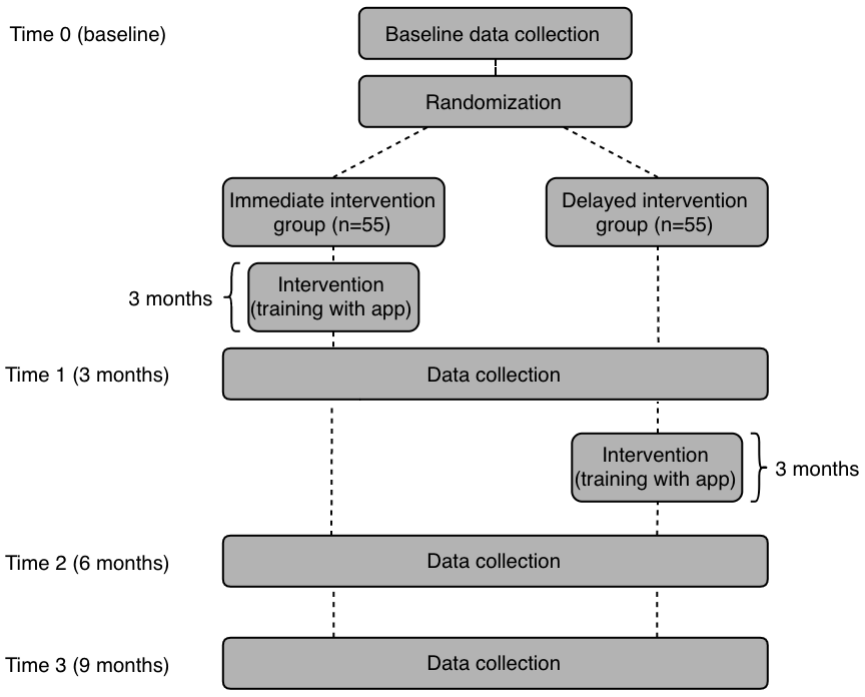
Study design.

This study will employ a mixture of qualitative and quantitative methods (Figure 1) [32]. The quantitative portion of the study will comprise a rater-blinded, randomized controlled trial with a stepped wedge design (ie, delayed intervention control group) [33]. This study design has been chosen over other study designs such as a randomized crossover controlled trial due to limitations in preventing carryover effect once participants have been exposed to the mobile app. The concern of potential carryover effect does not exist with a delayed intervention randomized controlled trial. A delayed intervention randomized controlled trial consists of 2 phases. In the first phase (first 3 months), participants are randomized to either have access to the app or not have access to the app (at this time point, they will serve as a control group that has received no intervention). This duration will allow for the effects of the self-management apps on our study outcomes (ie, self-management goals and improving participant self-efficacy) to be fully observed. In the second phase, all participants will have access to the mobile app for the remainder of the study. With this group, we will be able to see if the initial findings are replicated.

The qualitative portion will comprise semistructured interviews with a subsample of 1 in every 4 study participants. Embedding qualitative methods in randomized controlled trials has been suggested as a form of process evaluation [34,35]. Multiple methods will allow for data triangulation and increase the credibility of our findings [36]. This study will be documented according to the Consolidated Standards of Reporting Trials guidelines [37].

### Subject Recruitment

To be included in the study, participants need to (1) be living in the community, (2) have internet access, (3) be living in the community for at least 1 year after their injury, and (4) be able to provide their own consent. From our previous feasibility study, we know that most patients have either a tablet or access to a computer; however, we will purchase some tablets (n=10) for participants to borrow to be as inclusive as possible. We are currently working on making the app accessible via voice activation; however, we may need to exclude those who are unable to use either a computer or mobile device because of limited hand function. We anticipate including participants with spinal cord injury who have experienced a variety of different injury mechanisms and degrees of neurological impairment. Participants will be excluded if they (1) have previously used a self-management app focused on spinal cord injury, (2) are unable to communicate in English, or (3) have cognitive impairments that are likely to prevent them from reliably completing the study questionnaires, as identified using the 6-item cognitive impairment test (6-CIT; see procedures).

We will recruit participants from across the United States and Canada. We will connect and collaborate with other rehabilitation centers in the United States and Canada to facilitate recruitment through a variety of sources. Electronic means will include a study website that we will develop, a local rehabilitation institute’s website, e-blasts, and social media (eg, Facebook, Twitter). Participants will also be recruited by a local, spinal cord injury–focused, nonprofit organization’s peer recruitment coordinator through spinal cord injury support groups, via letters sent to former patients of a local rehabilitation center who meet the inclusion criteria, and previous research participants with spinal cord injuries who have given permission to be contacted about future research studies, via newspaper and newsletter advertisements. We will contact interested, potential participants by email or phone to confirm their eligibility. We will formally enroll eligible participants by obtaining their informed consent to participate. The risk to the safety of participants involved in the trial is minimal as the app is an alternative way to facilitate the development of self-management skills, an approach that has not been found to be detrimental in other populations. No adverse effects have been documented during pilot testing.

### Intervention

This individually tailored intervention will involve the use of a mobile app called “SCI Health Storylines” accompanied by 5 to 6 in-person, telephone, or Skype contacts that will occur over a 3-month period. At the beginning of the intervention, there will be 1 to 2 orientation sessions in which the principles of self-management will be reviewed; app-specific, self-management goals will be identified; features of the self-management app will be explained; and any access issues will be resolved. Over the first month, there will be 2 brief follow-up contacts, via telephone or Skype, to review any questions or issues participants are having with the app. During the last 2 months of the intervention, there will be monthly contacts to address the same issues.

SCI Health Storylines focuses on specific elements common to the management of spinal cord injury and uses key concepts such as goal setting and tracking of confidence in one’s ability to self-manage their condition [16]. We created tools to address the main self-management topics for individuals with spinal cord injury including bowel and bladder management, skin management, spasticity management, daily exercise, as well as more acute topics including urinary tract infections and autonomic dysreflexia. For each topic, the app prompts the user to set specific targets or goals and then allows the individual to journal their progress and self-management confidence. Generic (non-spinal cord injury specific) tools address other areas including medications and mood. The app is versatile and provides various options in how one wishes to track their self-management progress. For example, someone may select to enter their urine volumes after each catheterization, whereas others may opt to enter it at the end of the day.

Treatment fidelity will be promoted by providing in-depth training to the study interventionist. Treatment fidelity will be monitored by having the interventionists document elements of the intervention that they complete during each contact and by having the principal investigator or research coordinator monitor 5% of contact sessions throughout the research process. The benefits of such a client-patient-centered approach to care have been documented in similar settings and include increased adherence [38], improved clinical outcomes [39], improved communication between the client and provider, greater client satisfaction [38,39], and potentially increased cost-effectiveness [40].

### Primary Outcome Measure: Goal Attainment Scaling

Our primary outcome measure, goal attainment scaling, is a promising approach for evaluating psychosocial interventions in community settings (Table 1) [41]. This patient-centered measure will be used to identify self-management goals that participants want to achieve [42]. Goal attainment scaling has successfully been used to evaluate self-management goals in other clinical populations [43,44]. Goal attainment scaling is known to be sensitive and responsive to treatment and is ideal to use when no standardized measure accurately represents the goals and ideals of all participants [45]. With goal attainment scaling, objective outcomes are identified, which indicate degrees of attainment of participant-selected goals on a 5-point scale ranging from −2 to +2, where −2 is a much worse than expected outcome, 0 represents attaining the goal (the anticipated outcome), and 2 represents a much better than expected outcome; the aggregate T scores are then calculated. Test-retest reliability in nonclassroom educational study showed near-perfect correlation of goal attainment scaling when taken 3 days apart for 5 student-selected goals (Goal 1, *r*=1.000; Goal 2, *r*=.957; Goal 3, *r*=.953; Goal 4, *r*=.969; Goal 5, *r*=.749; *P*<.001) [46]. Goal attainment scaling has been found to be more sensitive than both the functional independence measure and the Barthel index [47] because standardized measures may not detect a change even when a goal is accomplished [48]. The minimal clinically important change for goal attainment scale is 10, based on the linear T score [49], which represents a change in score from the anticipated values.

To ensure that goals are measurable and outcomes are realistic, study personnel will be trained by 1 of the coauthors with experience using the measure to administer it in an objective manner [50]. Before beginning the intervention, participants will set approximately 6 self-management and app-related goals (minimum of 3) with a one-on-one remote trainer who is skilled in goal setting. The remote trainer will ensure that goals are specific, measurable, attainable (over a 3-month timeline), realistic, and defined in time (ie, SMART). Participants will weigh the goals by their relative importance [45]. As the degree of impairment of goals can depend on the degree of the spinal cord injury, this measure will not investigate the type or difficulty levels of self-selected goals. This measure will evaluate whether participants found they were able to achieve their individually tailored goals over the course of the intervention.

In addition, the question “How confident are you that you can achieve these goals?” has been added to the goal attainment scale to examine the self-efficacy of the participants. We have also added the question “How committed are you to achieving this goal?” to the goal attainment scale to examine participants’ goal striving and self-regulation, as this is related to self-efficacy.

### Secondary Outcome Measures

#### Self-Efficacy for Managing Chronic Disease Scale

The self-efficacy for managing chronic disease scale is designed to evaluate confidence in managing long-term disease [51,52]. It has been used extensively in many different populations including people with spinal cord injury to evaluate self-management interventions [53,54]. Each of the 6 items is rated on a scale of 1-10 (with 1 indicating “not at all confident” and 10 indicating “totally confident”), and an average score is calculated [52]. Lorig et al found that the scale was sensitive to their chronic disease self-management intervention [19]. Test-retest reliability was 0.72, and the minimal detectable change was found to be 2.25 among individuals with Parkinson disease [55].

#### Spinal Cord Injury Secondary Conditions Scale

The spinal cord injury secondary conditions scale targets secondary conditions related to spinal cord injury that have both direct and indirect impacts on health. The 16-item scale uses a 4-point ordinal scale (0-3) ranging from “no problem” to “significant problem,” with the total score ranging from 0-49. The test-retest reliability spinal cord injury secondary conditions scale has been measured at 5 different time points (at baseline, immediately post intervention, 4,8, and 12 months post intervention) and compared across each combination of time points. Findings from the study reveal that the spinal cord injury secondary conditions scale has adequate test-retest reliability between baseline and immediately post intervention (3 weeks after baseline; *r*=.698) [56]. Furthermore, this scale correlates highly with the 12-item Short-Form Health Survey (SF-12; Spearman *P* values range from .32 to .64) [56].

#### Self-Reported Health Care Utilization

Health care utilization will be measured by having participants record visits to see a physician, visits to hospital emergency departments, number of hospitalizations, and the number of nights spent in hospital [19]. Although there may be recall issues within self-reported health care utilization, it has been found to be highly correlated with days in hospital (*r*=.83) [13]. Participants will complete a weekly journal to help improve the accuracy of the report.

**Table 1:** Example of goal-attainment scaling for a person with autonomic dysreflexia.

Achieved (−2 to +2)		Behavioral statement of expected outcomes (over the course of 1 week)
**Yes**		
	Much better (+2)	Participant experiences 0 episodes of autonomic dysreflexia
	A little better (+1)	Participant experiences 1-3 episodes of autonomic dysreflexia
	As expected (0)	Participant experiences 4-5 episodes of autonomic dysreflexia
**No**		
	Same as baseline (−1)	Participant experiences 6 episodes of autonomic dysreflexia
	Worse (−2)	Participant experiences more than 6 episodes of autonomic dysreflexia

#### Self-Reported “Health Events” (Related to Self-Management of Spinal Cord Injury)

Participants will be asked to complete a weekly journal to document specific health events related to the key components of the self-management app (eg, urinary tract infections, episodes of autonomic dysreflexia, and pressure ulcers).

#### Health-Related Quality of Life

To measure general health status, health changes, and economic impact, participants will be asked to complete the EuroQol 5 Dimension 5 Level (EQ-5D-5L) survey. The EQ-5D-5L consists of 5 health-related dimensions including mobility, self-care, usual activities, pain or discomfort, and anxiety or depression [57]. Each of these dimensions contains 5 response items (no issue, slight issue, moderate issue, severe issue, and extreme issue). The response items (1-5) have no mathematical scoring system and cannot be interpreted as values on a cardinal scale. In addition, participants will also indicate their overall health status on a scale from 0 to 100 [57]. For test-retest reliability, minimal difference was found between administrations (0 and 14 days) of the measure in each of the 5 health dimensions with interclass coefficient scores ranging between .61 and .77 [58]. Furthermore, the scale correlates highly with the physical and mental component summary scores of the SF-12 (Spearman *P* values range from .41 to .67) [58].

#### App Usage

To understand the uptake of the intervention, basic analytics relating to the usage of the app will be evaluated, including the time spent logged into the app, the number of times logged into the app, and the specific features used within the app. This information will enable us to describe the uptake of the intervention and will complement the data obtained from the semistructured interviews.

#### Descriptive Information

Descriptive data will be collected about the participant’s sociodemographic characteristics such as age, sex, level of education, ethnic origin, language, marital status, type of dwelling, spinal cord injury etiology (traumatic or nontraumatic), lesion level, American Spinal Injury Association Impairment Scale, Spinal Cord Independence Measure 3 [59], and time since injury. In addition, the amount of informal and formal caregiving received (if any) will be recorded, and social support will be identified with the Interpersonal Support Evaluation List short form [60] and the Health Care Climate Questionnaire [61]. The method by which a patient can use the app will also be recorded (eg, manually, with a mouth stylus, and with the assistance of a caregiver). To assess the app’s overall quality across 4 dimensions (engagement, functionality, aesthetics, and information), participants will complete the Mobile Application Rating Scale [62]. We will also collect information on the amount of physical activity—at mild, moderate, and heavy intensity—the participants have performed over the previous 7 days using the Leisure Time Physical Activity Questionnaire for People with Spinal Cord Injury [63]. Finally, we will determine participants’ readiness to adopt new technologies using the technology readiness index [64].

#### Adherence

Adherence data will consist of the numbers of goal attainment–focused orientation sessions participants complete with the interventionist, the length of each session, and participants’ app usage. Specific data participants enter into the app will also be recorded to further describe app usage patterns. This information will allow us to describe the uptake of the experimental intervention and which aspects of self-management participants specifically focus on. This information will complement the data collected from semistructured interviews.

#### Treatment Fidelity

To ensure treatment fidelity, we will follow the strategies and guidelines outlined by Lenker et al and Borrelli et al [65,66]. The experimental intervention will be conducted by interventionists trained by the principal investigator to use the same standardized approach and materials as described in a treatment manual. Interventionists will follow and complete a checklist to note the completion of each step in the study protocol. Throughout the study, interventionists will also be observed performing the intervention by the principal investigator to ensure adherence with the study protocols.

### Procedures

**Figure 2:**
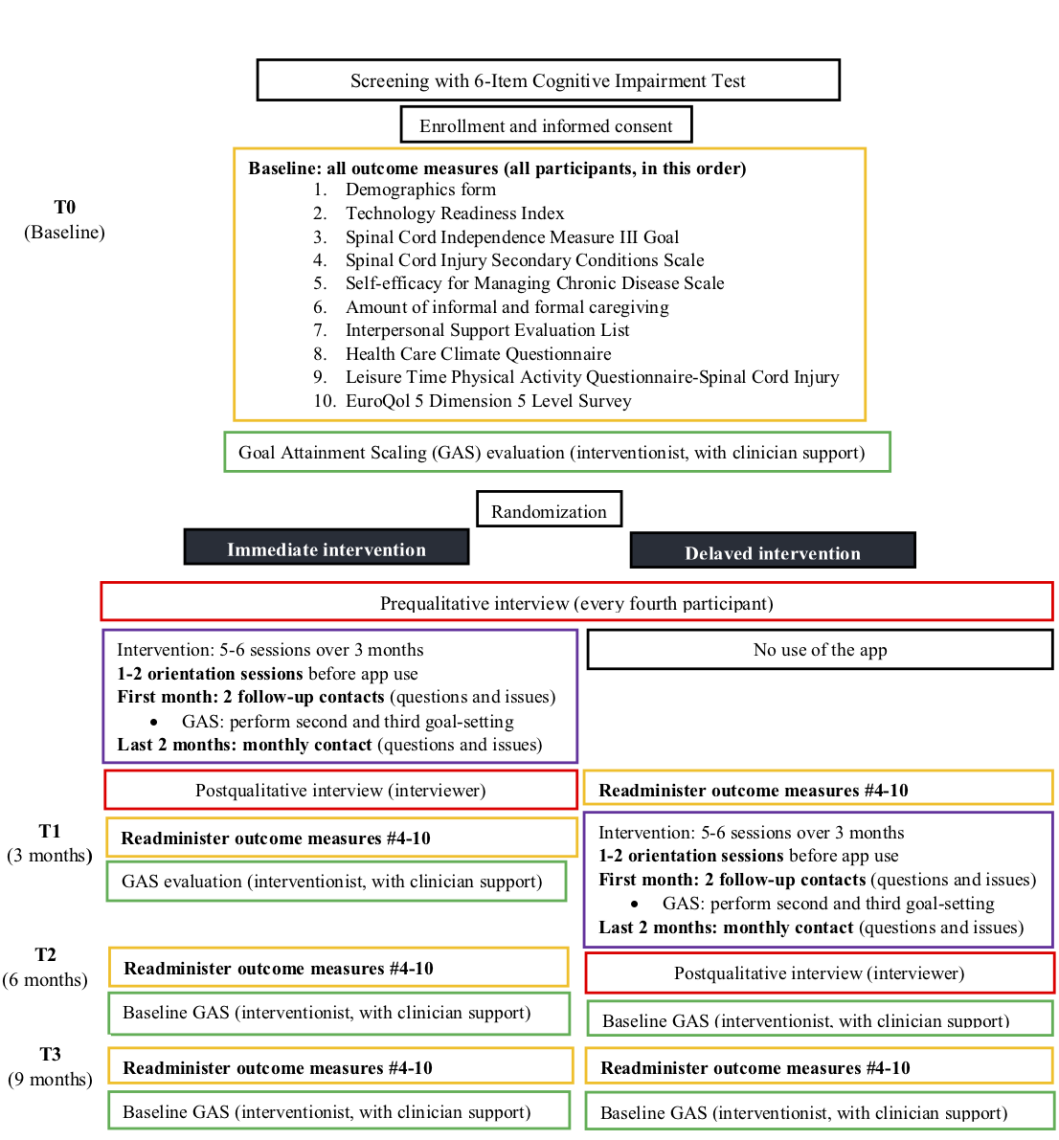
Detailed overview of the study procedures.

We will use a Web-based randomization program that will be managed by the project manager. Participants will be randomized to receive the intervention either immediately after baseline data are collected or after a 3-month delay via a randomization service (Figure 2). Participants in both groups will be asked to refrain from using any other self-management apps for the duration of the study. Three months into the intervention (T1), we will compare the outcomes from the immediate intervention group with those from the delayed intervention group. Participant outcomes at 6 months (T2) and 9 months (T3) into the intervention will allow us to determine how well outcomes are maintained in the immediate intervention group, if the effects of the intervention are replicated in the delayed intervention group, and to determine longer-term effects of the intervention.

Potential participants will be screened using the 6-CIT [67]. If participants score above the inverse cut-off score of 7, they will be excluded from the study. After screening, informed consent will be obtained, and sociodemographic data and baseline measures will be collected from all participants. Data will be collected for participants at baseline (T0), at 3 months (T1), at 6 months (T2), and at 9 months (T3). Participants will be given the option of completing the questionnaires themselves through a link supplied by the Qualtrics Survey Platform (Qualtrics, Provo, Utah, United States of America) or over the phone, through a rater (research assistant). To protect participant privacy, we will use H.264 encryption on the mobile device and on mobile app servers.

T1 will be considered the primary end point for the intervention, but T3 will be considered the final end point for the study. As it is not possible to blind the participants, the interventionist, or the qualitative interviewer, a single blind study design will be employed in which data collectors are blinded to participants’ group allocation. The delayed intervention group will complete the baseline measures at the same time but will delay the intervention by 3 months. For the delayed intervention group, T1 will occur before the intervention and T2 will occur immediately following the intervention. Figure 2 provides a detailed overview of the study procedures.

Data collection for all participants will take between 5.5 and 7.5 hours. Time spent doing the intervention will vary depending on app usage, which should range from 10 hours (for those using the app for only a few minutes per day) to more than 60 hours (for those in the immediate intervention group who use the app for 30 min per day). Therefore, the total time commitment will range from 15.5 to 67.5 hours, depending on the desired app usage of the participant.

### Qualitative Interviews

When attempting to interpret research findings, researchers often report personal retrospective impressions about the intervention. The quality of this research material is debatable, as it typically is not methodically collected [68]. To better understand how the self-management intervention was experienced by participants and administered by clinicians, a series of 2 qualitative semistructured interviews will be conducted at baseline (T0) and following the intervention (T1 for the immediate intervention group and T2 for the delayed intervention group) with a subsample of study participants. These qualitative methods will help facilitate a deeper understanding of the quantitative findings, especially if they are divergent between participants [69], and will not interfere with the intervention or with the acquisition of quantitative data. At baseline, participants will be asked questions about (1) their current self-management strategies living with spinal cord injury, (2) their experiences (including challenges) with self-management, and (3) their expectations of the intervention. After the intervention, we will explore how the intervention was experienced by participants and will identify barriers and facilitators to improve intervention implementation in the future. This will include questions about (1) their overall impression of the intervention they received, (2) things they liked and disliked about the intervention, (3) how the intervention could be improved, and (4) recommendations for implementation. The preliminary interview guide is provided in Textbox 1.

The qualitative portion of the study will be conducted primarily by a single research assistant to limit the potential effects of change in characteristics and settings found with involving too many interviewers. The research assistant will know each participant’s group allocation (delayed or immediate). This will allow for the qualitative interviews to be corroborated with the quantitative results. If the interviewer is not available to perform interviews, a replacement interviewer will be available to conduct qualitative interviews. To help build consistency between both interviewers, a semistructured interview guide will help both interviewers focus on their discussion. Furthermore, each interviewer will have undergone a practice session with the principal investigator to ensure they have received proper and equivalent training in conducting one-on-one interviews. Each interviewer’s first 2 to 3 interview sessions will be observed by the principal investigator to provide constructive feedback and ensure consistency.

Qualitative interviews will be voice-recorded using a digital recorder and transcribed verbatim by a research assistant. After being transcribed, the transcripts will be reviewed while listening to the recorded audio file to ensure accuracy. Interview field notes that describe the nonverbal behavior and impressions of participants, and the influence of the interviewer on data collection [70], will also be recorded.

### Sample Size

To calculate the sample size for the delayed intervention randomized controlled trial portion of the study, we used the formula described by Diggle et al with T1 as the primary end point for the intervention [69]. The sample size was based on an estimated effect size of 0.58 (Cohen *d*) from a previous intervention study that used goal attainment scaling as an outcome measure [50]. With an assumed correlation of .7 between T0 and T1, the required sample size is 46 participants per group (92 in total). Given a possible dropout and premature withdrawal rate of 20%, a sample of 110 participants will be recruited [71]. Our goal is to reach the target sample size in approximately 2 years. If our target sample size cannot be reached within this time frame, we will continue to recruit individuals until our target sample size can be reached.

For our qualitative analysis, we will use sequential sampling and interview every fourth participant in the study in each group (immediate intervention and delayed intervention). This will enable us to get a robust representative sample of participants that should be large enough to achieve data saturation [72,73].

### Quantitative Data Analysis

A generalized linear mixed-effect model (GLMM) will be used to assess the association between treatment (immediate intervention vs delayed intervention) and primary and secondary outcomes. GLMM has a number of advantages over other approaches to analyzing longitudinal data, as cases with missing data are not excluded, and so, no imputation is required; it can also handle unbalanced time points while incorporating all data [74,75]. Secondary outcome analyses will be considered exploratory, given the likelihood for increased type 1 errors that result from multiple statistical comparisons. All statistical analysis will be completed with IBM SPSS Statistics Version 22 [76].

### Treatment Fidelity Analysis

The percentage of intervention protocol items completed for each subject will be calculated for each participant to determine the treatment fidelity. These quantitative data will be supplemented with data from the qualitative portion of the study.

### Qualitative Data Analysis

A thematic analysis will be conducted on the qualitative data using the 5-step process outlined by Braun and Clark [77]. The data analysis will be performed using NVivo 10 (QRS International, Victoria, Australia). As an additional means to monitor treatment fidelity, a content analysis will be performed. Results from the treatment reflections and qualitative interviews will be compared with the quantitative results to identify divergent and complementary findings. Qualitative data will also be compared with quantitative data for individual participants.

**Interview guide.**

Baseline

- How would you describe your health currently?
- How do you currently manage your health (ie, living with a spinal cord injury)?
- How satisfied are you with the way you currently manage your health?
- What, if any, self-management challenges do you currently experience?
- What are your self-management goals?
- How do you feel about using a mobile self-management health app for your self-management?
- What, if any, worries or concerns do you have about using the self-management app?
- How do you imagine the self-management app will change your daily life?
- What are you hoping to learn from participating in this study?

After the intervention

- What did you think about the mobile self-management health app?
- How easy was the app for you to use?
- How, if at all, did the app help you attain your self-management goals? a. What were the challenges?
- How confident were you at achieving your goals?
- How, if at all, did the app help manage your secondary complications from your spinal cord injury?
- What was your favorite “tool” and why?
- What was your least favorite “tool” and why?
- What was your overall impression of the intervention you received?
- Which aspects of the intervention did you find most helpful?
- What did you think about how much time was required? a. For example, for learning to use the app, for recording your updates?
- What did you think about the timing of the intervention?
- How much impact, if any, on achieving your self-management goals do you attribute to the self-management app?
- How much impact, if any, on achieving your self-management goals do you attribute to other factors?
- What, if any, benefits did you experience from the intervention?
- Were there any problems that you encountered? a. If yes: Do you have any suggestions for solving the problems?
- How would you describe your health now?
- Do you have any additional comments you would like to add?

### Soundness of the Research

A randomized controlled trial is the best way to evaluate the efficacy of a clinical intervention [78]. Given our inability to blind participants to the intervention they will receive, we will implement single blinding, in which raters are blinded to participants’ group allocation. For the qualitative data, we will use reflexivity, triangulation, and member checking to help ensure the trustworthiness of the analyses and findings [79]. We will make interview notes and memos to serve as reflexive tools [77]. This will also help to detail the analytic processes. Multiple data sources and methods of collection will be used for data triangulation, thus increasing the credibility of the findings [36]. Member checking will also be conducted to allow participants the opportunity to review the preliminary study findings and provide feedback about the conclusions made from the data.

### Limitations

Although we intend to recruit individuals with a variety of different spinal cord injuries, including individuals with high and low tetraplegia, we do not currently have the resources to create an app with voice activation. In our pilot study, some participants used a mouth stylus or relied on caregivers to enter information into the app. Furthermore, individuals with high tetraplegia will be able to use a large-sized tablet or mobile phone for performing daily tasks on the self-management app with little difficulty.

Another limitation of this study is our ability to determine the active ingredients of the intervention. We can evaluate this to some extent because we track app usage. We will be able to perform a subanalysis to explore whether adherence is related to primary or secondary outcomes. Furthermore, we will ask participants to comment on how much they feel the app is responsible for achieving their primary and secondary outcomes during qualitative interviews. However, we will not be able to determine the relative contribution of different aspects of the intervention on participants’ outcomes.

## Results

### Anticipated Results

By encouraging individuals with spinal cord injury to adopt positive health behaviors and promoting their autonomy, we intend to demonstrate that SCI Health Storylines can help people with spinal cord injury attain their self-management goals, improve their self-efficacy, reduce secondary complications, and decrease health care utilization.

### Study Timeline

A 3-year study timeline is presented in Table 2. In the first 6 months (Q4 2017 and Q1 2018), we obtained research ethics and hired and trained research staff (health professionals who will administer the intervention and research assistants who will collect the data). We will now recruit and collect data over the next 24 months. We will analyze the data and conduct knowledge translation projects over the last 6 months of the study.

**Table 2:** Project timeline.

Item	2017	2018				2019				2020		
	Q4	Q1	Q2	Q3	Q4	Q1	Q2	Q3	Q4	Q1	Q2	Q3
Obtain research ethics	✓											
Hire and train staff	✓	✓										
Hire and train health professionals	✓	✓										
Recruit participants		✓	✓	✓	✓	✓	✓	✓	✓			
Provision of interventions		✓	✓	✓	✓	✓	✓	✓	✓			
Data collection		✓	✓	✓	✓	✓	✓	✓	✓	✓		
Quantitative data analysis										✓	✓	
Qualitative data analysis				✓	✓	✓	✓	✓	✓	✓	✓	
Knowledge translation									✓	✓	✓	✓

## Discussion

Self-management apps have been shown to enhance self-management of chronic conditions such as diabetes and asthma [11,15,16]. As yet, no research has been conducted to determine if this is true for people with spinal cord injury. Having an effective self-management app that is generic (ie, can be used to promote self-management in a variety of areas) and not resource-intense (ie, it is self-directed and does not have extensive formalized involvement of peer mentors and health care professionals) would be extremely beneficial as it would enable people with spinal cord injury to learn the fundamental skills of self-management more independently (eg, goal setting, problem solving, symptom tracking and management, skill development, and self-efficacy) as a form of secondary prevention [80].

If the results are positive, this study will produce credible new knowledge describing multiple outcomes that people with spinal cord injury realize from an app-based self-management intervention. Ultimately, reducing secondary complications will greatly improve the quality of life of people with spinal cord injuries and will reduce health care costs.

## Abbreviations

6-CIT: 6-item cognitive impairment test
EQ-5D-5L: EuroQol 5 Dimension 5 Level
GLMM: generalized linear mixed-effect model
SF-12: 12-item Short-Form Health Survey

## Appendix

Multimedia Appendix 1 Craig H. Nielsen Foundation peer-review comments.
