# Supplementary material for: Improving Self-Management Skills Among People With Spinal Cord Injury: Protocol for a Mixed-Methods Study
Source: JMIR Res Protoc. 2018 Nov 14;7(11):e11069. doi: 10.2196/11069 (PMC6301832; doi:10.2196/11069)
Supplement: Multimedia Appendix 1 [file resprot_v7i11e11069_app1.pdf]

Proposal Information

Applicant: Mortenson, Ben
Title Improving Self-Management Skills among People with Spinal Cord Injury
Type/Competition PSR Studies and Demonstration Projects
Institution University of British Columbia
App # 440872

Committee: PSR 2017 FGA

Summary Statement

Text Admin Summary:
The Neilsen Foundation has conditionally approved funding of this proposal. Please see the reviewer summaries for a discussion of strengths and weaknesses. The following issue(s) deserve particular attention: This study is one of the few randomized control trials to be undertaken for e-health interventions to improve self-care management for those with SCI. Enthusiasm for the study to test affordable and replicable app tools was driven by the innovative design, the excellent research environment and the clear analysis plan developed by the PI. Concerns with adequate powering and probable recruitment issues should be addressed early to ensure successful completion of the study.

Reviewer Role: Primary (237823)

OVERALL IMPACT
After considering all of the review criteria, summarize the significant strengths and weaknesses of the application. In this funding category, does this project address important gaps or propose cutting-edge ideas, interventions and/or test approaches that have great potential to have a positive impact for those living with SCI? Please state the likelihood that the project scope suits this funding level and will develop an area of work that has the potential to exert a sustained powerful influence on the SCI field and/or be an important contributor to the field of SCI research.

:
This is an interesting application which proposes a study on improving self- management skills among people with SCI. The goal is to evaluate the efficacy of a broad based self-management application. The application is innovative but the study is methodologically weak. There are no hypotheses listed and no psychologist are involved with the study. Attrition and data tracking are concerns.

SIGNIFICANCE
1. Does the project address an important problem or a critical barrier in the field?

2. If the Aims of the project are achieved, how would this work change or enhance current methods, technologies, treatments, services, or interventions?

:
Averting secondary conditions after SCI is an important issue and thus helping to develop self-management skills to do is can make a significant contribution to the field. The literature review provided is very good, up to date and comprehensive. The project objective is clearly described and although not identified as so, there are appear to be 4-5 aims listed under the objective. The attainment of these aims could inform future interventions in SCI.

RELEVANCE
1. How is this project relevant to the mission of the Neilsen Foundation?

2. How is this project relevant to the goal of the Psychosocial Research portfolio?

:
The project and training is relevant to the CHN Foundation mission as it relates to the improvement of the wellbeing and quality of life of persons with SCI. The aims are also consistent with the PSR program goals and portfolio.

INVESTIGATOR(S)
1. Are the PI, collaborators, and other contributors well suited to the project?

2. If the PI is a junior investigator, does he/she have appropriate experience, training and facilities to do the proposed work? If the PI is an established investigator, has he/she demonstrated an ongoing record of accomplishments that have advanced their field(s)?

:
The PI is an Assistant Professor in the Department of Occupational Therapy and is well positioned to conduct the proposed research. He has relevant research experience and publications. His team of investigators and support personnel appear also strong but the application could benefit from adding a psychologist to the group to address self-management issues.

INNOVATION
1. Does the project challenge and seek to shift current research or clinical practice/program intervention paradigms by utilizing novel theoretical concepts, approaches or methodologies, instrumentation, or interventions?

2. And/or does this application apply concepts, approaches or methodologies, instrumentation, or interventions from another field of research to spinal cord injury?

3. And/or is a refinement, improvement, or new application of theoretical concepts, approaches or methodologies, instrumentation, or interventions proposed?

:
The project is somewhat innovative as it proposes to evaluate the efficacy of an app designed to improve self-management skills among persons with SCI living in the community. If efficacious this intervention can inform future treatments and CPG for those living with SCI.

APPROACH
1. Are the overall strategy, methodology, and analyses well-reasoned and appropriate to accomplish the specific aims of the project, within the proposed project period and by the project team described? Are potential problems, alternative strategies, and benchmarks for success presented?

2. Are preliminary data provided to support the feasibility of the project? Or, if the project is in the early stages of development, will the strategy establish feasibility and will particularly risky aspects be managed adequately?

3. If the project involves clinical and/or community-based research: A) are the plans for protection of human subjects from research risks described and adequate; and B) are the plans for recruitment of patients/participants appropriate?

:
The approach looks very good. They propose to use us a mixed method design. They propose to use a RCT design as well further enhancing the strength of this proposed methodology. Concerns include: 1) insufficient number of contacts towards the last two months of the intervention; this would be an excellent stage to collect more data on the use of this app and its outcomes; 2) potential problems with recruitment and attrition as they may loose subjects on the delayed arm– some type of unrelated intervention may keep subjects interested in participating without adding to effects; 3) not sure how recruitment from another country (US) will influence this approach since distance and healthcare cultures are different – this needs clarification; 4) recruitment could be challenging due to attrition (n=46 for power calculations) – not clear how many will participate in the qualitative phase of this study – a table summarizing subject recruitment for both methods would have improved reader's understanding; 5) some of the measures are not SCI validated and for the sample size there appears to be too many variables and measures; 6) exposure in terms of hours to the app will vary widely – how differences attributed to this variation will be treated is not clear. Preliminary data provided supports the feasibility of this project. There is no discussion of safety issues or subjects' protection.

ENVIRONMENT
1. Will the institutional environment in which the work will be done contribute to the probability of success?

2. Are the institutional support, physical equipment and other resources available to the investigators adequate for the project proposed?

3. Will the project benefit from unique features of their environment, subject populations, or collaborative arrangements?

:
The environment is excellent (ICORD and RH) and very conducive of this research. Recruitment of participants as noted earlier could become an issue. No other concerns.

NON-SCORED CRITERIA
(PLEASE NOTE THAT THE SCORE FOR THIS NON-SCORED SECTION MUST BE A "50-NONSCORED." PLEASE DISREGARD SCORING OPTIONS 1-9 FOR THIS SECTION ONLY.)

Please provide any important or relevant comments on each of the non-scored criterion below.

1. Budget

2. Ethics/Safety

3. Other/Additional Comments for the Applicant

4. RESUBMISSION: When reviewing a Resubmission, the committee will evaluate the application as now presented, taking into consideration the responses to comments from the previous scientific review group and changes made to the project.

:
Budget – Seems appropriate for the scope of this project. There are no incentives for participants. This may help recruiting and retaining subjects.
Ethics/Safety – This needs to be clarified as it is not discussed by the applicant.

Reviewer Role: Secondary (138888)

OVERALL IMPACT
After considering all of the review criteria, summarize the significant strengths and weaknesses of the application. In this funding category, does this project address important gaps or propose cutting-edge ideas, interventions and/or test approaches that have great potential to have a positive impact for those living with SCI? Please state the likelihood that the project scope suits this funding level and will develop an area of work that has the potential to exert a sustained powerful influence on the SCI field and/or be an important contributor to the field of SCI research.

:
Key strengths of the proposal are that the self-management app targets a wide range of health behaviors that can affect overall quality of life. They plan a RCT (delayed intervention controls) design with qualitative and quantitative elements. They use blinded outcome assessments. They have already developed the app and gotten positive feedback from clinicians and people with SCI including have people actually use it and continued to use it after the initial study period. The measurement plan is extremely thoroughly described and comprehensive. The only weakness is that they mysteriously based their effect size on a study that is totally unrelated to their intervention but happens to use their primary outcome. As a result the study probably will be under-powered. Still, they plan to have enough subjects that is should provide relatively accurate and rich pilot data for a definitive trial.

SIGNIFICANCE
1. Does the project address an important problem or a critical barrier in the field?

2. If the Aims of the project are achieved, how would this work change or enhance current methods, technologies, treatments, services, or interventions?

:
Yes, a app/eHealth approach to improving self-management, they target a wide range of self-management topics so the intervention could benefit a large number of people with SCI

RELEVANCE
1. How is this project relevant to the mission of the Neilsen Foundation?

2. How is this project relevant to the goal of the Psychosocial Research portfolio?

:
It aims to improve QOL via improved self-management of SCI related conditions which is reasonable and logical

INVESTIGATOR(S)
1. Are the PI, collaborators, and other contributors well suited to the project?

2. If the PI is a junior investigator, does he/she have appropriate experience, training and facilities to do the proposed work? If the PI is an established investigator, has he/she demonstrated an ongoing record of accomplishments that have advanced their field(s)?

:
Look like an excellent team with strong pilot work and expertise in the areas needed including RCTs and qualitative research

INNOVATION
1. Does the project challenge and seek to shift current research or clinical practice/program intervention paradigms by utilizing novel theoretical concepts, approaches or methodologies, instrumentation, or interventions?

2. And/or does this application apply concepts, approaches or methodologies, instrumentation, or interventions from another field of research to spinal cord injury?

3. And/or is a refinement, improvement, or new application of theoretical concepts, approaches or methodologies, instrumentation, or interventions proposed?

:
Yes it takes a reasonably large incremental step towards testing a suite of apps to support self-management, they have sufficient pilot data to take this next step.

APPROACH
1. Are the overall strategy, methodology, and analyses well-reasoned and appropriate to accomplish the specific aims of the project, within the proposed project period and by the project team described? Are potential problems, alternative strategies, and benchmarks for success presented?

2. Are preliminary data provided to support the feasibility of the project? Or, if the project is in the early stages of development, will the strategy establish feasibility and will particularly risky aspects be managed adequately?

3. If the project involves clinical and/or community-based research: A) are the plans for protection of human subjects from research risks described and adequate; and B) are the plans for recruitment of patients/participants appropriate?

:
Very thorough and detailed application. Everything is described precisely. With just one exception the methods are outstanding. The only weakness I found was a moderate one, the power analysis. They base their effect size on a seemingly random study that used GAS as an outcome in infants with motor delays. The effect size is huge (.58) an allows them to claim their study is adequately powered. I think it would have been more reasonable to report what the effect sizes are of self-management trials or app related health behavior change interventions--there must be a meta-analysis or two on these topics. Effect sizes from those sources would be more believable. As a result the study is likely to be significantly under-powered. One remedy is for the study to be conceptualized as a pilot RCT. It is large enough that it could lead to effect-size estimates for future studies that might be fairly accurate.

ENVIRONMENT
1. Will the institutional environment in which the work will be done contribute to the probability of success?

2. Are the institutional support, physical equipment and other resources available to the investigators adequate for the project proposed?

3. Will the project benefit from unique features of their environment, subject populations, or collaborative arrangements?

:
Seems excellent. The team is centered at UBC/GF Strong. They have collaborative relationship with the Rick Hansen Institute

NON-SCORED CRITERIA
(PLEASE NOTE THAT THE SCORE FOR THIS NON-SCORED SECTION MUST BE A "50-NONSCORED." PLEASE DISREGARD SCORING OPTIONS 1-9 FOR THIS SECTION ONLY.)

Please provide any important or relevant comments on each of the non-scored criterion below.

1. Budget

2. Ethics/Safety

3. Other/Additional Comments for the Applicant

4. RESUBMISSION: When reviewing a Resubmission, the committee will evaluate the application as now presented, taking into consideration the responses to comments from the previous scientific review group and changes made to the project.

:
No response entered

©2007-2016 Altum, Inc. All rights reserved	Contact Us	Terms of Service	Acceptable Use Policy	Privacy Policy
